# Xylem plasticity of root, stem, and branch in *Cunninghamia lanceolata* under drought stress: implications for whole-plant hydraulic integrity

**DOI:** 10.3389/fpls.2024.1308360

**Published:** 2024-02-19

**Authors:** Shubin Li, Xiaoyan Huang, Ruping Zheng, Maxiao Zhang, Zhiguang Zou, Kate V. Heal, Lili Zhou

**Affiliations:** ^1^ Forestry College, Fujian Agriculture and Forestry University, Fuzhou, China; ^2^ Chinese Fir Engineering Technology Research Center of the State Forestry and Grassland Administration, Fuzhou, China; ^3^ Huaying Forestry Development Center, Huaying, China; ^4^ College of Resources and Environment, Fujian Agriculture and Forestry University, Fuzhou, China; ^5^ School of Geo Sciences, University of Edinburgh, Edinburgh, United Kingdom; ^6^ College of Geography and Oceanography, Minjiang University, Fuzhou, China

**Keywords:** drought stress, *Cunninghamia lanceolata*, hydraulic properties, anatomical structure, xylem plasticity, trade-off strategy, pot experiment

## Abstract

**Introduction:**

A better understanding of xylem hydraulic characteristics in trees is critical to elucidate the mechanisms of forest decline and tree mortality from water deficit. As well as temperate forests and forests growing in arid regions, subtropical and tropical forests are also predicted to experience an increased frequency and intensity of climate change-induced drought in the near future.

**Methods:**

In this study, 1-year-old *Cunninghamia lanceolata* seedlings (a typical subtropical species in southern China) were selected for a continuous controlled drought pot experiment of 45 days duration. The experimental treatments were non-drought (control), light drought, moderate drought and severe drought stress, which were 80%, 60%, 50%, and 40%, respectively of soil field maximum moisture capacity.

**Results:**

The hydraulic conductivity, specific conductivity and water potential of roots, stems, and branches of *C. lanceolata* all decreased with the prolonging of drought in the different drought intensities. The relative decrease in these hydraulic values were greater in roots than in stems and branches, indicating that roots are more sensitive to drought. Root tracheid diameters normally reduce to ensure security of water transport with prolonged drought, whilst the tracheid diameters of stems and branches expand initially to ensure water transport and then decrease to reduce the risk of embolism with continuing drought duration. The pit membrane diameter of roots, stems and branches generally increased to different extents during the 15–45 days drought duration, which is conducive to enhanced radial water transport ability. The tracheid density and pit density of stems generally decreased during drought stress, which decreased water transport efficiency and increased embolism occurrence. Correlation analysis indicated that anatomical plasticity greatly influenced the hydraulic properties, whilst the relationships varied among different organs. In roots, tracheid diameter decreased and tracheid density increased to enhance water transport security; stems and branches may increase tracheid diameter and pit membrane diameter to increase hydraulic conductivity ability, but may increase the occurrence of xylem embolism.

**Discussion:**

In summary, under drought stress, the xylem anatomical characteristics of *C. lanceolata* organs were highly plastic to regulate water transport vertically and radially to maintain the trade-off between hydraulic conductivity efficiency and safety.

## 1 Introduction

Drought stress is likely increasing with climate warming in many temperate and tropical forests (IPCC, 2007), and increased frequency of extreme drought events has resulted in widespread tree mortality and forest timber yield decline (Blackman et al., 2019; Wang et al., 2021). Hydraulic failure resulting in xylem embolism has been reported as a major reason for tree mortality under anomalous drought (Körner, 2019; Bittencourt et al., 2020). Trees can improve drought adaptation through regulating the xylem hydraulic structure and water transport strategy (Tramontini et al., 2013; Anderegg and Meinzer, 2015). The hydraulic conductivity (*K*_h_) and specific conductivity (*K*_s_) in shoots decreased in water-stressed plants, which were helpful in reducing water loss to the atmosphere (Lovisolo and Schubert, 1998; Ladjal et al., 2005). Many studies found that root hydraulic traits varied greatly among different drought tolerant species, with drought-sensitive trees apparently having larger mean vessel diameters in roots to increase water transport capacity (Köcher et al., 2012; Losso et al., 2016). However, previous studies were focused either on above-ground organs (stems or branches) or on below-ground roots. Plant hydraulic systems are composed of a network of vessels or tracheids in the xylem that enables the continuous water supply from roots to shoots (Rodriguez-Dominguez et al., 2018; Chen et al., 2020). Understanding the hydraulic properties in the integrated whole-plant water transport system is essential for predicting the water flow in the soil-plant-atmosphere continuum and improving plant drought resistance (Domec et al., 2017).

According to the Hagen-Poiseuille equation, hydraulic efficiency increases exponentially with increasing vessel or tracheid diameter (Tyree and Zimmermann, 2002). However, larger vessels increase the risk of hydraulic failure due to embolism resulting from significant lower pressure in the xylem under drought stress (Cruiziat et al., 2002; Hajek et al., 2014). Long-term drought stress is expected to decrease vessel or tracheid diameter and increase vessel or tracheid density in xylem in the trunk to enhance hydraulic efficiency and hydraulic safety under drought stress for many plants (Lovisolo and Schubert, 1998; Falchi et al., 2020). The response of xylem hydraulic transport to drought depends on xylem anatomy, and xylem anatomical plasticity during water deficit also varies among different organs and different drought intensities (Köcher et al., 2012). About half of plants’ total hydraulic resistance is located belowground, and xylem in roots is more prone to cavitation than that in shoots (Alder et al., 1996; Pockman and Sperry, 2000; Martínez-Vilalta et al., 2002). However, it remains unclear how drought stress affects anatomical characteristics and how the anatomy affects the xylem hydraulic properties between roots, stems and branches. The vertical and radial hydraulic transport from roots to stems/branches needs to be examined simultaneously to increase our understanding of the plant hydraulic mechanisms in response to water deficiency.

Although numerous studies have focused on arid and semi-arid plants, limited attention has been paid to the drought adaptation and response of woody species in subtropical humid or sub-humid regions (Dichio et al., 1999; Rzepecki et al., 2011; Zhou et al., 2013). In recent years, due to climate change, spatial variability in precipitation and seasonal droughts have resulted in increased frequency of forest mortality in temperate, tropical and subtropical regions, for example, *Eucalyptus urophylla* forest and rain forest species (Zhou et al., 2011; Trueba et al., 2017; Chen et al., 2020). Trees in arid environments may develop a series of survival strategies, such as transferring non-structural carbohydrates to roots or sacrificing more vulnerable and expendable organs, to ensure the integrity of root hydraulic function and optimize the ability to recover from embolism after rehydration (Rodriguez-Dominguez et al., 2018). However, it may be expected that due to the lack of drought adaption, trees growing in humid regions are more likely to die of hydraulic transport failure caused by drought.

Chinese fir [*Cunninghamia lanceolata* (Lamb.) Hook.] is one of the most important evergreen conifer species in southern China, with a planted area of 17 × 10^6^ ha that accounts for approximately 24% of forest plantations in China and 6.1% globally (FAO, 2015; Liu et al., 2018). In China, *C. lanceolata* is distributed across 17 provinces and autonomous regions, ranging from 21° 31′ to 34° 03′ N, and from 101° 30′ to 121° 53′ E. The large geographical range of Chinese fir means it has a high risk of exposure to drought stress caused by the increasing spatial and seasonal variability of precipitation associated with global warming, especially due to subtropical high pressure in the Pacific Ocean (Li et al., 2020a). Drought effects on the growth and survival of Chinese fir are particularly devastating because drought stress usually occurs in summer and autumn in southern China, which is the time of most rapid growth of the species (Martínez-Vilalta et al., 2002; Lin et al., 2004). However, there are significant knowledge gaps in understanding the drought response mechanisms of Chinese fir in southern China. Studies to date have focused on screening for drought-tolerant genotypes, and the effects of drought on tree growth, physiology and biochemistry (Lin et al., 2004; Li et al., 2020a; Li et al., 2020b), but there are still few studies on the hydraulic transport characteristics and anatomy in the xylem of Chinese fir during drought stress.

Our previous research showed that root hydraulic properties of Chinese fir varied among different diameter classes and were affected by both tracheid and pit structures (Huang et al., 2023). However, the effects of drought stress on the vertical hydraulic conductance from root to stem, and pit anatomical characteristics within each organ still need to be clarified. In view of this, we hypothesized that: (1) hydraulic properties vary greatly among different organs (root, stem and branch), and specifically that the hydraulic response of roots is more sensitive to drought than that of stems and branches; (2) xylem anatomical structure is plastic in response to different degrees of drought stress, and this plasticity varies among different organs; and (3) there are strong relationships between hydraulic properties and anatomical characteristics of tracheids and pits in xylem under different drought stress intensities. To test these hypotheses, we determined hydraulic parameters and anatomy in xylem in roots, stems and branches under different soil water deficit levels in 1-year-old Chinese fir seedlings. This allowed us to identify how the xylem anatomical plasticity in different organs regulates hydraulic conductivity of Chinese fir to adapt to drought stress.

## 2 Materials and methods

### 2.1 Plant materials and sampling

The experiment was conducted in the Intensive Breeding Greenhouse of the Chinese Fir Engineering Technology Research Center, National Forestry and Grass Bureau of Fujian Agriculture and Forestry University. The materials were 1-year-old Chinese fir seedlings grown from third-generation seeds of Chinese fir species at the tree nursery in Youxi National Farm, Fujian Province, China. In early July 2021, 100 saplings of *C. lanceolata* were planted in individual pots (~30 cm diameter and 35 cm high). After 3 months of cultivation with gravimetric soil water content approximately equal to maximum soil field water capacity (44.83%), we chose healthy, straight, and well-grown saplings (~40 cm high and 0.50 cm basal diameter) for the drought stress experiment. The experiment lasted 45 days during October and November, selected to coincide with the second of the annual growth peaks in Chinese fir. During the experiment, the temperature in the greenhouse was in the range 24 - 31°C, with an 11.5 hour photoperiod under daylight conditions. The seedlings were allocated randomly among the following four treatments: control (CK), light drought (LS), moderate drought (MS), and severe drought (SS), which approximated to 80%, 60%, 50% and 40% of the maximum soil field capacity (44.83%), respectively. The CK treatment was irrigated with tap water once every 2–3 days, and the amount of water over time was maintained at 80% of maximum field water capacity. The drought treatment was created by not watering during the experimental set-up to achieve the desired soil water, and then maintained at the prescribed soil water content by watering if required. Soil moisture was measured daily using time-domain-reflectometry sensors (TZS-2X, China) to maintain soil water content at the prescribed value. All the drought stress treatments were carried out for 45 days, with 4 seedlings selected at random from each drought treatment for destructive sampling at 0, 15, 30, and 45 days.

At each sampling time, the Chinese fir seedlings were completely removed from the pot to ensure that the roots were not damaged. The seedlings were immediately placed in a black plastic bag and put in a water-filled bucket, then returned quickly to the laboratory to prevent evaporation of water. Next, the seedlings were cut into stem, branch, and root segments, which were placed in water for 30–60 min for dark adaptation, cut again underwater, and the cuts at both ends were trimmed flat with a razor blade. All stem, branch, and root segments were cut to a similar length of ~ 4 cm. The mean diameters of stem, first-order twig (representing branch) and coarse root segments were 6.0 ± 0.2, 2.5 ± 0.5 and 2.8 ± 0.5 mm, respectively.

### 2.2 Measurements of hydraulic properties

Hydraulic traits were measured in four segments each of root, stem and branches for each treatment at each sampling time, according to Hajek et al. (2014) using an Xylem apparatus (XYL’EM-Plus, France). The total number of segments measured were 192 (3 organs × 4 seedlings × 4 treatments × 4 sampling times). The segment section was flushed with 0.03 mol L^-1^ KCl solution at low pressure (0.01 MPa) and the water flow was measured (*F*, kg min^-1^). The hydraulic conductivity (*K_h_*, g·m·MPa·^-1^·min^-1^) of each organ segment was calculated by multiplying the water flow mass per minute with the sample length (m) and dividing by the pressure (MPa m^-1^). The specific conductivity (*K_s_*, g·m·MPa^-1^ min^-1^·cm^-2^) of each organ segment was calculated by dividing the hydraulic conductivity by the cross-sectional area (cm^2^) of the sample sapwood. The hydraulic conductivity per unit mass (*C_um_*, m·MPa^-1^·min^-1^) was calculated by dividing the hydraulic conductivity by the dry weight (g) of each organ segment. Leaf specific conductivity (LSC, g·m·MPa^-1^ min^-1^·g^-2^) was calculated by dividing the hydraulic conductivity by the dry weight (g) of the leaves at the end of the stem segment. Huber values (*H_v_*, cm^2^·g^-1^) were calculated by dividing the cross-sectional area of the sapwood per stem segment by the dry weight of the leaves at the end of the stem segment. Water potential was determined using a dew point water potential meter (WP4C, America) (Pan et al., 2020; Li et al., 2022).

### 2.3 Measurements of anatomical structure

The stem, branch, and root segments from the same sample, which were cut to determine hydraulic properties, were chosen to determine anatomical structure. The total number of segments were 192 (3 organs × 4 seedlings × 4 treatments × 4 sampling times). Each segment was cut into strips of 0.5 cm thickness with a razor blade, and stored in FAA fixative (acetic acid: ethanol: formalin = 1:1:1) for 24 h. Subsequently, thin sections were created by softening, dehydration, embedding, sectioning, staining, and gluing processes (Hajek et al., 2014). Photographs were taken at ×400 magnification using a biomicroscope (Nikon ECLIPSE E200, Japan) to obtain transverse and longitudinal sections. The tracheid diameter (*D*, μm), pit membrane diameter (*P*_d_, μm), and pit aperture diameter (*A*_d_, μm) were calculated using Image J software (http://rsb.info.nih.gov/ij). Other anatomical properties were calculated according to the following formulae: tracheid density (*N*, 10^3^ mm^-2^) = tracheid numbers/the transverse section area; pit density (*N*_pm_, 10^3^ mm ^-2^) = pit numbers/the longitudinal section area; pit open ratio (*R*_ap_) = pit aperture diameter/pit membrane diameter.

### 2.4 Measurements of xylem density

Firstly, five segments of root, stem and branch approximately 4 cm long were cut for each sampling, and the volume (V, cm^3^) of the fresh segments was measured by the water volume displacement method. Secondly, the fresh samples were dried in an oven at 75°C for 72 h to a constant weight. Finally, the dry mass (M, g) was measured using a balance with an accuracy of 0.0001 g. Xylem density (*Dx*, g cm^-3^) was calculated as dry mass/fresh volume (Pan et al., 2020).

### 2.5 Statistical analysis

Two-way analysis of variance (ANOVA) was used to assess any differences in xylem hydraulic and anatomical properties for each organ with the drought stress intensity and duration as the principal factors. One-way ANOVA was performed to compare the differences in hydraulic and anatomical traits among different drought intensities within each sampling time and among different sampling times within each drought intensity treatment, followed by the least significant difference (LSD) test at *P* < 0.05. All data were tested with the Kolmogorov-Smirnov test for normality and the Levene test for the homoscedasticity assumption before further analyses.

When constant variance was not satisfied, a log or square transformation was used. Pearson’s correlation analysis was performed to study the relationships between hydraulic and anatomical properties among roots, stems and branches within different drought stress intensities. All statistical analyses were conducted using SPSS Statistical Package (SPSS 17.0, SPSS Inst., IL, USA). Figures were plotted using Origin 2021 (OriginLab Corp., Northampton, MA, USA).

## 3 Results

### 3.1 Effects of drought stress on the hydraulic properties

**Figure 1:**
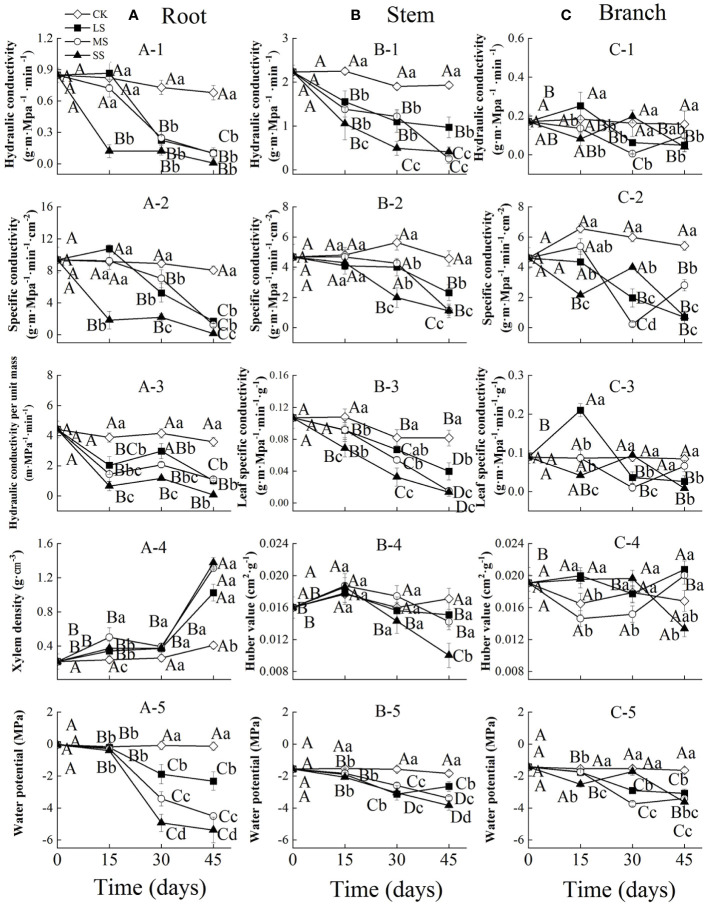
Xylem hydraulic properties in the root **(A)**, stem **(B)** and branch **(C)** of 1-year-old Chinese fir seedlings with different drought durations (0–45 days). **(A-1)**: root hydraulic conductivity, **(A-2)**: root specific conductivity, **(A-3)**: root hydraulic conductivity per unit mass, **(A-4)**: root xylem density, (A-5): root water potential; **(B-1)**: stem hydraulic conductivity, **(B-2)**: stem specific conductivity, **(B-3)**: stem leaf specific conductivity, **(B-4)**: stem Huber value, **(B-5)**: stem water potential; **(C-1)**: branch hydraulic conductivity, **(C-2)**: branch specific conductivity, **(C-3)**: branch leaf specific conductivity, **(C-4)**: branch Huber value, **(C-5)**: branch water potential. Treatments: CK, control (no-drought stress); LS, light drought stress; MS, moderate drought stress; SS, severe drought stress. Note different y-axis scales between segments for some properties. Different upper-case letters indicate significant difference among sampling times within each drought intensity treatment. Different lower-case letters indicate significant difference among drought intensities within each sampling time. The differences were compared using ANOVA *post hoc* means with least significant difference (LSD) analysis (*P* < 0.05). Values are means ± standard error (n = 4).

Two-way ANOVA indicated that drought stress intensity, stress duration and their interactions had significant effects on hydraulic properties in roots (**Table 1**). The root hydraulic conductivity and specific conductivity in the severe drought treatment decreased significantly during drought stress, while these values changed slightly after 15 days and then decreased significantly in the light and moderate drought treatments with prolonged drought. The hydraulic conductivity per unit mass of roots decreased significantly after 15 days, then increased slightly at 30 days and decreased at 45 days across all drought intensities. The xylem density of roots remained stable from 0 to 30 days, and then increased significantly in all treatments. The water potential of roots was stable from 0 to 15 days, but decreased significantly from 15 to 45 days among different drought intensities. At 15 days, severe drought stress significantly decreased the root hydraulic conductivity and specific conductivity. In contrast, at 30 days, water potential significantly decreased with increasing drought intensities (**Figure 1A**).

**Table 1:** Two-way ANOVA of drought stress intensity (df = 3) and drought stress duration (df = 3), and their interactions on xylem hydraulic properties of 1-year-old Chinese fir seedlings.

Plant organ and property	Drought stress intensity		Drought stress duration		Drought stress intensity × Drought stress duration	
	*F*	*P*	*F*	*P*	*F*	*P*
Root						
Hydraulic conductivity	67.206	< 0.001***	132.616	< 0.001***	16.796	< 0.001***
Specific conductivity	66.843	< 0.001***	103.380	< 0.001***	17.869	< 0.001***
Hydraulic conductivity per unit	34.159	< 0.001***	53.917	< 0.001***	4.293	< 0.001***
Xylem density	29.945	< 0.001***	182.663	< 0.001***	13.889	< 0.001***
Water potential	504.226	< 0.001***	944.457	< 0.001***	149.269	< 0.001***
Stem						
Hydraulic conductivity	37.093	< 0.001***	62.685	< 0.001***	5.965	< 0.001***
Specific conductivity	28.417	< 0.001***	70.885	< 0.001***	11.664	< 0.001***
Leaf specific conductivity	44.587	< 0.001***	156.376	< 0.001***	8.806	< 0.001***
Huber value	2.279	0.091	9.335	< 0.001***	2.099	0.048*
Water potential	117.016	< 0.001***	261.355	< 0.001***	32.473	< 0.001***
Branch						
Hydraulic conductivity	19.917	< 0.001***	45.575	< 0.001***	29.154	< 0.001***
Specific conductivity	28.143	< 0.001***	51.744	< 0.001***	21.923	< 0.001***
Leaf specific conductivity	32.666	< 0.001***	82.887	< 0.001***	55.191	< 0.001***
Huber value	3.148	0.033*	1.748	**0.170**	4.553	< 0.001***
Water potential	120.699	< 0.001***	313.780	< 0.001***	65.830	< 0.001***

*significant at P < 0.05; ***significant at P < 0.001.

Drought stress intensity, duration and their interactions had significant effects on hydraulic properties in stems (except for the effects of intensity on Huber values) (**Table 1**). The stem hydraulic conductivity and leaf specific conductivity both showed a significantly decreasing trend in different drought treatments during drought stress. The specific conductivity and Huber values of stems remained stable initially and then decreased significantly from 15 to 45 days for different drought intensities. The water potential of stems generally decreased significantly in all treatments with prolonged drought, and differed significantly between drought treatments at 30 and 45 days (**Figure 1B**).

The branch hydraulic properties were also greatly influenced by drought stress intensity, duration and their interactions (except for the effects of duration on Huber values) (**Table 1**). The branch hydraulic conductivity and leaf specific conductivity values fluctuated substantially between different drought intensities and durations. The specific conductance of branches showed a decreasing trend over time in the light drought treatment, and fluctuating change in moderate and severe drought stress. Branch Huber values did not change significantly with increasing drought duration under moderate and severe drought, but increased significantly under light drought. The water potential of branches generally decreased in all treatments with increasing drought duration, and differed significantly among drought intensities at each (**Figure 1C**).

### 3.2 Effects of drought stress on the anatomical structure

**Figure 2:**
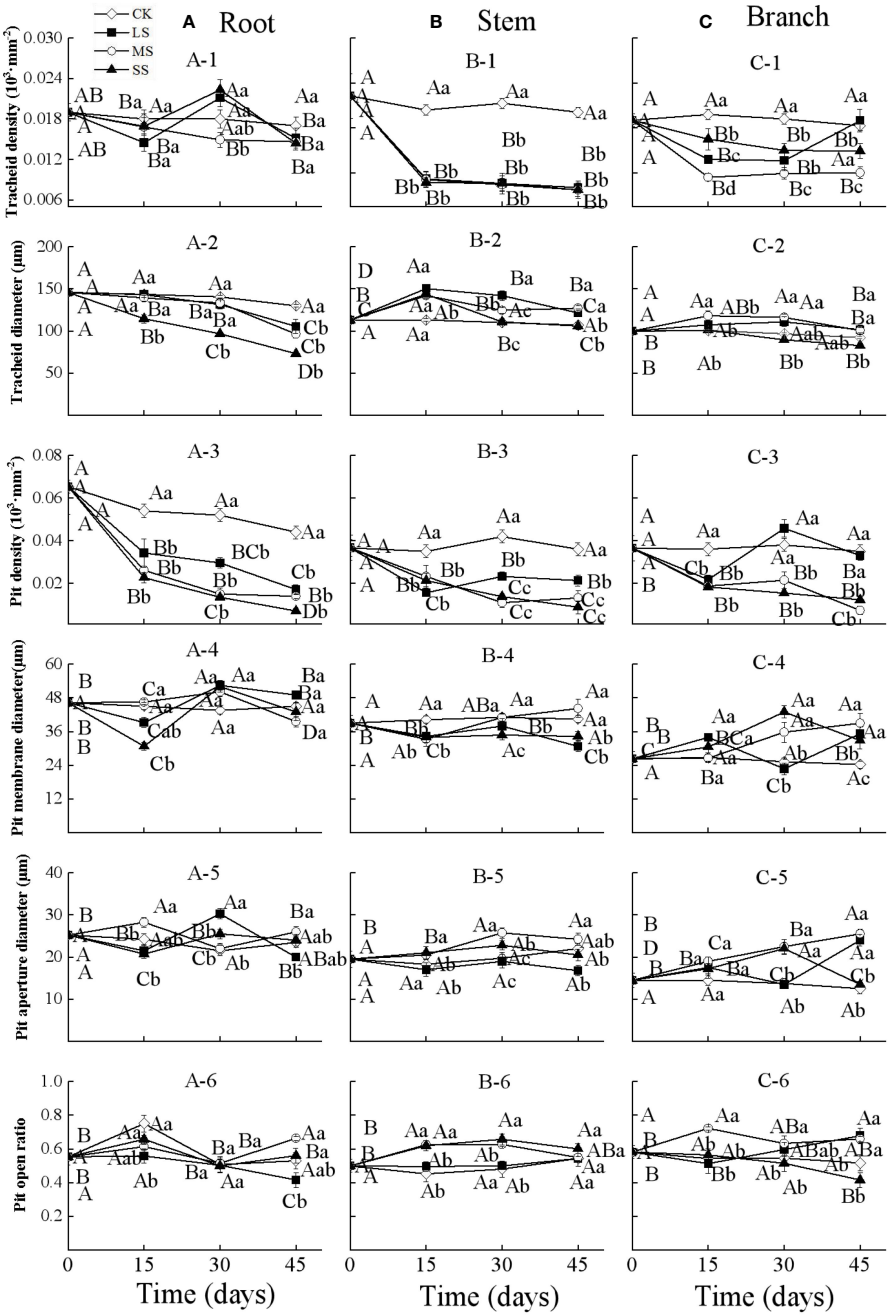
Xylem anatomical properties in the root **(A)**, stem **(B)** and branch **(C)** of 1-year-old Chinese fir seedlings with different drought durations (0–45 days). **(A-1)**: root tracheid density, **(A-2)**: root tracheid diameter, **(A-3)**: root pit density, **(A-4)**: root pit membrane diameter, **(A-5)**: root pit aperture diameter, **(A-6)**: root pit open ratio; **(B-1)**: stem tracheid density, **(B-2)**: stem tracheid diameter, **(B-3)**: stem pit density, **(B-4)**: stem pit membrane diameter, **(B-5)**: stem pit aperture diameter, **(B-6)**: stem pit open ratio; **(C-1)**: branch tracheid density, **(C-2)**: branch tracheid diameter, **(C-3)**: branch pit density, **(C-4)**: branch pit membrane diameter, **(C-5)**: branch pit aperture diameter, **(C-6)**: branch pit open ratio. Treatments: CK, control (no-drought stress); LS, light drought stress; MS, moderate drought stress; SS, severe drought stress. Note different y-axis scales between segments for some properties. Different upper-case letters indicate significant difference among sampling times within each drought intensity treatment. Different lower-case letters indicate significant difference among drought intensities within each sampling time. The differences were compared using ANOVA *post hoc* means with least significant difference (LSD) analysis (*P* < 0.05). Values are means ± standard error (n = 4).

Two-way ANOVA indicated that drought stress intensity, duration, and their interactions had significant effects on tracheid diameter and pit density in roots (**Table 2**). The tracheid density of roots showed a fluctuating increasing trend during 30 days in the light and severe drought treatments, whilst for the moderate drought, it decreased significantly as drought duration increased. For all drought intensities, the root tracheid diameter and pit density both decreased significantly with drought duration, and the values were all lower than control treatments at each sampling time. The root pit membrane diameter decreased at 15 days, and then fluctuated over time, with significant differences between treatments occurring after 15 days. The root pit aperture diameter fluctuated substantially between different drought intensities and durations, but significantly varied between drought intensities at each timestep. The root pit open ratio for different drought treatments fluctuated over time and the differences were not significant at each drought timestep sampling (**Figure 2A**).

**Table 2:** Two-way ANOVA of the effect of drought stress intensities (df = 3) and drought stress duration (df = 3), and their interactions on xylem anatomical properties of 1-year-old Chinese fir seedlings.

Plant organ and property	Drought stress intensity		Drought stress duration		Drought stress intensity × Drought stress duration	
	*F*	*P*	*F*	*P*	*F*	*P*
Root						
Tracheid density	2.070	0.117	15.568	< 0.001***	4.190	< 0.001***
Tracheid diameter	50.911	< 0.001***	97.312	< 0.001***	8.263	< 0.001***
Pit density	30.883	< 0.001***	83.399	< 0.001***	3.756	0.001***
Pit membrane diameter	1.566	0.210	9.376	< 0.001***	3.898	0.001***
Pit aperture diameter	1.159	0.335	1.548	0.214	5.117	< 0.001***
Pit open ratio	1.974	0.130	2.132	0.108	0.971	0.476
Stem						
Tracheid density	77.058	< 0.001***	116.366	< 0.001***	8.682	< 0.001***
Tracheid diameter	132.621	< 0.001***	187.350	< 0.001***	34.860	< 0.001***
Pit density	77.904	< 0.001***	70.666	< 0.001***	15.214	< 0.001***
Pit membrane diameter	12.764	< 0.001***	5.362	0.003**	5.366	< 0.001***
Pit aperture diameter	13.180	< 0.001***	5.674	0.002**	2.879	0.008**
Pit open ratio	18.081	< 0.001***	6.678	0.001**	4.268	< 0.001***
Branch						
Tracheid density	62.361	< 0.001***	40.946	< 0.001***	13.166	< 0.001***
Tracheid diameter	31.230	< 0.001***	17.901	< 0.001***	5.327	< 0.001***
Pit density	34.141	< 0.001***	22.098	< 0.001***	7.732	< 0.001***
Pit membrane diameter	25.742	< 0.001***	19.839	< 0.001***	19.127	< 0.001***
Pit aperture diameter	32.569	< 0.001***	15.935	< 0.001***	17.269	< 0.001***
Pit open ratio	17.885	< 0.001***	0.361	0.781	6.463	< 0.001***

**significant at P < 0.01; ***significant at P < 0.001.

Drought stress intensity, duration, and their interactions had significant effects on xylem anatomical properties in stems and branches (except for the effects of durations on pit open ratio in branch) (**Table 2**). The tracheid density and the pit density of stems decreased significantly at 15 days and then changed slightly for the remainder of the experiment. The tracheid diameters of stems increased significantly after 15 days and then decreased with drought prolonging for all treatments; values were significantly different at each sampling time. The pit membrane diameter of stems decreased at 15 days and then increased with significant differences between drought intensities at 30 days. The pit open ratio of stems did not change significantly with drought duration under light drought stress, but increased significantly with drought duration in both the moderate and severe drought treatments (**Figure 2B**).

The tracheid density and the pit density of branches decreased significantly after 15 days for all the treatments, and then continuously decreased for the severe drought treatments. The tracheid diameters of branches significantly increased after 15 days and then returned to initial values under light and moderate drought, whilst the values under severe drought stress significantly decreased from 15 to 45 days. The pit membrane diameter of branches increased generally with drought duration in the moderate and severe drought stress treatments, while fluctuating under light drought stress. The pit aperture diameter of branches generally showed an increasing trend with drought duration under light and moderate drought stress, whilst the values increased from 0 to 30 days, then decreased significantly for the severe drought stress. The pit open ratio of branches generally increased with drought duration under light and moderate drought stress, and decreased under severe drought stress (**Figure 2C**).

### 3.3 Correlation analysis between hydraulic properties and anatomy

Associations between hydraulic properties and anatomical structure were analyzed using Pearson correlation coefficients. The hydraulic conductivity, specific conductivity, hydraulic conductivity per unit mass, and water potential of roots were significantly and positively correlated with tracheid diameter and pit density. Xylem densities of roots were significantly inversely correlated with tracheid diameter, tracheid density and pit density (**Table 3**). For stems, hydraulic conductivity, specific conductivity, leaf specific conductivity, and water potential were significantly positively correlated with tracheid density and pit density. Hydraulic conductivity, leaf specific conductivity, and water potential were significantly positively correlated with pit density. Conversely, these hydraulic values were significantly negatively correlated with pit aperture diameter and with pit open ratio. For branches, specific conductivity and water potential were significantly positively correlated with tracheid density and pit density. Furthermore, these hydraulic values were negatively correlated with pit membrane diameter and pit aperture diameter (**Table 4**).

**Table 3:** Pearson bivariate correlations between the xylem hydraulic structure and its anatomy in roots of 1-year-old Chinese fir seedlings.

Plant organ	Anatomical property	Hydraulic conductivity	Specific conductivity	Xylem density	Hydraulic conductivity per unit mass	Water potential
Root	Tracheid density	0.129	0.119	-0.496^**^	0.277*	0.125
	Tracheid diameter	0.807^**^	0.881^**^	-0.779^**^	0.709^**^	0.826^**^
	Pit density	0.761^**^	0.712^**^	-0.629**	0.794^**^	0.750^**^
	Pit membrane diameter	0.060	0.189	-0.131	0.151	-0.144
	Pit aperture diameter	0.008	-0.008	-0.065	0.132	-0.040
	Pit open ratio	-0.021	-0.093	0.072	-0.097	0.034

*significant at P < 0.05; **significant at P < 0.01.

**Table 4:** Pearson bivariate correlations between the xylem hydraulic structure and its anatomy in stems and branches of 1-year-old *C. lanceolata* seedlings.

Plant organ	Anatomical property	Hydraulic conductivity	Specific conductivity	Leaf specific conductivity	Huber value	Water potential
Stem	Tracheid density	0.856**	0.627**	0.735**	0.104	0.744**
	Tracheid diameter	-0.243	0.103	-0.020	0.348**	-0.067
	Pit density	0.791**	0.668**	0.750**	0.250*	0.781**
	Pit membrane diameter	0.244	0.197	0.086	-0.087	0.137
	Pit aperture diameter	-0.316*	-0.207	-0.434**	-0.140	-0.273*
	Pit open ratio	-0.545**	-0.384**	-0.542**	-0.092	-0.399**
Branch	Tracheid density	0.345**	0.287*	0.042	0.167	0.542**
	Tracheid diameter	-0.143	-0.017	0.054	-0.015	-0.073
	Pit density	0.189	0.259*	0.043	0.043	0.425**
	Pit membrane diameter	-0.112	-0.431**	-0.033	0.133	-0.448**
	Pit aperture diameter	-0.239	-0.406^**^	-0.138	0.258*	-0.472**
	Pit open ratio	-0.175	-0.026	-0.181	0.216	-0.088

*significant at P < 0.05; **significant at P < 0.01.

## 4 Discussion

### 4.1 Hydraulic structures of plant organs are plastic in drought conditions

Our results verified our first hypothesis that the plant hydraulic system can respond plastically to drought, but the hydraulic response varied among different organs for Chinese fir (**Figure 1**). For control seedlings, the hydraulic conductivity (*K_h_*) was in the order stem > root >branch, and the specific conductance (*K_s_*) of roots was higher than that of stems and branches, while the *K_h_* and *K_s_* of roots, stems, and branches all gradually decreased with drought duration. This demonstrated that water transport capacity in *C. lanceolata* was reduced under drought stress, similar to *Oryza sativa* L. and *Quercus ilex* (Limousin et al., 2010; Tabassum et al., 2016). However, *Populus euphratica* and *Pinus ponderosa* were reported to have enhanced *K_h_* under drought stress (Maherali and DeLucia, 2000; Li et al., 2019). These differences are probably due to species-specific hydraulic characteristics of plants in arid and sub-tropical regions (Hukin et al., 2005). The *K_h_* reflects the water transport ability in xylem, while *K_s_* and hydraulic conductivity per unit mass are important indicators of water transport efficiency (Schumann et al., 2019). With *K_h_* decreasing in the xylem, cavitation would occur when metastable liquid water is replaced by water vapor due to the expansion of gas bubbles, which form an embolism or air blockage that disrupts water transport (Lens et al., 2013). We found that the *K_s_* of the roots, stems and branches in xylem under severe drought stress decreased by 98.6%, 76.3%% and 84.1%, respectively, after 45 days (**Figures 1A-2, B-2, C-2**), which indicated that roots may be more susceptible to the risk of hydraulic failure in water-stressed conditions, and break the soil-plant-atmosphere continuum (Ayup et al., 2012; Johnson et al., 2018). We also found that leaf specific conductivity (LSC) of stems decreased more markedly than branches with the prolongation of drought, which demonstrated that branches of Chinese fir have more resilience in long-distance water transport to meet the water requirement of distal leaves (Hacke et al., 2015).

Drought stress can directly affect the water status of plants, with water potential representing an important indicator of water deficit that reflects the extent of plant drought stress (Makbul et al., 2011; Sun et al., 2020). Under water stress, xylem water potential in plants decreases and the xylem tension increases, which is another major cause of embolization in the xylem vessel or tracheid (Liu et al., 2021). In this study, the water potential of roots decreased markedly from -0.05 MPa to -5.38 MPa after 45 days of severe drought stress, and the decrease degree was greater than that of the stems and branches (**Figures 1A-5, B-5, C-5**). This is consistent with our results of the *K_h_* and *K_s_* in the roots decreasing more markedly than those in stems and branches, which again verified our first hypothesis that the hydraulic response of roots may be more sensitive to drought. Roots are the first organs to have contact with soil water and thus perceive the occurrence of soil drought. The lower xylem water potential in roots under drought facilitates water uptake from the soil, but increases the susceptibility of roots to embolization or cavitation. In our previous study, we found that larger roots (diameter > 5 mm) have lower water potential than smaller diameter classes (< 2 mm and 2-5 mm) in Chinese fir (Huang et al., 2023), indicating that larger diameter roots may be more prone to embolism due to higher xylem tension.

Plants may develop varied drought tolerance strategies including increasing water uptake and reducing water loss (Ogasa et al., 2013). Root resistance to drought and water uptake ability can enable plants to recover from drought, even following catastrophic damage to the shoots, depending on the physiological functions of different-sized roots and adaptation ability to environmental stress (Tsuda and Tyree, 1997). Furthermore, plants could decrease water loss by adjusting stomatal conductance under water deficit conditions, which can maintain a relatively constant leaf water potential, thereby reducing drought damage and tree mortality (Alder et al., 1996; Li et al., 2013). Some studies also find plants can increase non-structural carbohydrates (NSC) concentrations and soluble sugar content in all tissues under severe drought, thereby reducing water potential, maintaining cell expansion, and increasing water uptake under drought conditions (Wang et al., 2021).

### 4.2 Anatomical structures of plant organs are plastic in drought conditions

The study results verified our second hypothesis that the xylem anatomical structure was plastic and the plasticity varied among plant organs under drought stress. Plants uptake and transport water from soil to stem vertically, which is strongly affected and plastic by vessel or tracheid structure in response to drought (Jacobsen et al., 2012; Oladi et al., 2014). Chinese fir is a gymnospermous plant, which transports water upward through tracheids. In this study, the tracheid diameter in roots tended to decrease with drought duration, while the tracheid diameter in both stems and branches tended to increase in the early drought stage and then decrease in the later drought stage (**Figures 2A-2, B-2, C-2**). According to the Hagen-Poiseuille equation, water flow rate increased exponentially with the xylem vessel diameter (Hajek et al., 2014), indicating that narrow xylem vessel diameter decreases hydraulic conductivity ability, and decreases the occurrence of xylem embolism (Hukin et al., 2005). Plants may regulate xylem anatomy to maintain a trade-off between hydraulic efficiency and hydraulic safety under drought stress. We found that the decreased tracheid diameter in roots under drought stress may enhance the resistance to cavitation compared to aboveground organs (stems and branches). However, the hydraulic efficiency was not only related to vessel diameter, but also to vessel density in xylem (Hajek et al., 2014). In our study, after 15 days of drought stress, the tracheid density of roots, stems, and branches all showed a general decreasing trend, which was most significant in the stems and branches (**Figures 2A-1, B-1, C-1**). In order to compensate for the decreased water transport caused by the sharp change in the tracheid density, the tracheid diameter of stems and branches increased greatly (Falchi et al., 2020; Lübbe et al., 2022).

Water transport occurs not only vertically through vessels, but also radially through the bordered pits of vessels (Hacke and Sperry, 2001). Although we did not directly measure the radial conductivity in xylem, the pit anatomical characteristics can also reflect the radial hydraulic conductivity. Pit membranes in gymnosperms function as safety valves during water transport and protect layers from external threats such as air bubbles (Hacke et al., 2015; Li et al., 2022). It has been shown that gymnosperms adapt to drought by increasing pit membrane diameter, pit aperture diameter, and pit open ratio in stems and branches during drought, which is helpful for radial water transport (Lens et al., 2011; Han et al., 2022). In the present study, pit membrane diameter in roots and stems increased in response to drought stress from 15 to 30 days (**Figures 2A-4, B-4, C-4**), which enhances water flow radially between different tracheids. This result is consistent with previous studies that reported increased pit membrane diameter of *Pseudolarix amabili*, *Cunninghamia lanceolata* and *Cedrus deodara* (Han et al., 2022) and increased perforation and pit diameters in stem xylem of *Populus euphratica* Oliv. under drought stress (Xu and Chen, 2012). However, in our study, pit density generally showed a decreasing trend in all organs with increasing drought duration, accompanied by decreased tracheid diameter in roots (**Figures 2A-2, A-3, B-3, C-3**), resulting in decreased water transfer efficiency of roots. The pit density in the newly formed tracheids under drought stress is plastic to regulate radial hydraulic transport. According to the efficiency-safety trade-off theory, this anatomical plasticity in roots of Chinese fir would decrease hydraulic efficiency and support hydraulic safety.

### 4.3 How the anatomical structures affects hydraulic properties among different organs

Our third hypothesis – that drought stress changes the hydraulic structure of Chinese fir by affecting its tracheid and pit structure – was confirmed by the strong correlations between hydraulic properties and anatomical characteristics in xylem under different drought stress intensities. In roots, hydraulic conductivity, specific conductivity, hydraulic conductivity per unit mass, and water potential were highly significantly positively correlated with tracheid diameter and pit density (**Table 3**). The hydraulic conductivity of stems and branches was highly significantly positively correlated with tracheid density (**Table 4**). Other studies have also shown that plant xylem tracheid number and inter-tracheid pit characteristics are closely related to xylem hydraulic conductivity (Lens et al., 2011; Shafqat et al., 2021). Woodrum et al. (2003) reported that species cavitation pressure was strongly correlated with inter-vessel pit structure (membrane thickness and porosity), weakly correlated with pit number per vessel, and not related to pit area per vessel. It has been demonstrated that water transport efficiency is promoted by greater tracheid diameter and tracheid density, and larger pit membrane diameter and higher pit density in xylem (Pan et al., 2020; Han et al., 2022). Under drought stress in the current study, *C. lanceolata* organs adopted different drought-tolerance mechanisms. These included decreasing tracheid diameter and pit density and increasing wood density in roots to enhance water transport safety vertically. In addition, pit membrane diameter of roots, stems and branches increased to different extents at various drought durations to increase water transport efficiency radially.

## 5 Conclusion

We have demonstrated that xylem hydraulic properties in different *C. lanceolata* organs can be regulated by anatomical plasticity in response to drought stress. The hydraulic conductivity, specific conductivity, and water potential in roots, stems and branches all decreased with drought duration. The tracheid diameter and pit density in roots decreased with drought duration, and tracheid diameter and pit membrane diameter generally increased in stems and branches under prolonged drought stress. The risk of embolism was reduced by decreasing the tracheid diameter and increasing xylem density to ensure the safety of root water transport. Water transport efficiency was enhanced by increasing tracheid diameter and pit membrane diameter in order to satisfy the water demand of aboveground organs. The hydraulic properties were highly related to anatomical structure in xylem of *C. lanceolata*, but the correlations varied greatly among different organs. The response of hydraulic and anatomical properties in xylem of *C. lanceolata* under drought were plastic, to maintain a balanced trade-off between water transport efficiency and hydraulic safety. The plasticity response to drought between anatomical structures and hydraulic properties in trees in subtropical areas may differ compared to those in temperate and arid areas. Thus, tropical and subtropical forests may die of hydraulic imbalance due to the lack of adaptation capacity to long-term drought. Although our study was carried out in a controlled greenhouse experiment with seedlings, it has provided an important new quantitative understanding of the drought resistance mechanism of subtropical plants. Further work will be ongoing to ascertain if these mechanisms also operate in field conditions.

## Data availability statement

The original contributions presented in the study are included in the article. Further inquiries can be directed to the corresponding author.

## Author contributions

SL: Conceptualization, Funding acquisition, Methodology, Supervision, Writing – review & editing. XH: Formal Analysis, Investigation, Resources, Software, Writing – original draft. RZ: Investigation, Methodology, Resources, Writing – review & editing. MZ: Formal Analysis, Investigation, Resources, Writing – review & editing. ZZ: Formal Analysis, Investigation, Software, Writing – original draft. KH: Formal Analysis, Software, Writing – review & editing. LZ: Conceptualization, Formal Analysis, Funding acquisition, Investigation, Writing – original draft.
